# The Anti-Vivisection Hospital

**Published:** 1910-01-08

**Authors:** 


### THE ANTI-VIVISECTION HOSPITAL.

### NEGLECT TWICE FOUND BY JURY WITH THE CORONER'S WARNING.

*First Case.*

At Battersea on Tuesday Mr. John Troutbeck held an inquest on the body of Elizabeth Styan (56), late of Granfield Street, Battersea. She had been employed in the laundry at Wandsworth Infirmary, and while at work she ran a pin into her thumb. Blood poisoning resulted, and she died at the infirmary on Christmas Day. Soon after she received the injury she went to the Anti-Vivisection Hospital at Battersea. It was stated by her son and daughter that she saw no doctor there, though she waited nearly two hours. A nurse bathed her hand and kept it in a bowl a long time. The patient nearly fainted.

The Coroner said that the Anti-Vivisection Hospital called itself a general hospital, whatever that might precisely mean. In connection with other hospitals one associated the term with certain facts, such as the continual attendance of a regular staff. That was the second case which had come to his knowledge recently where no medical man had been at the hospital when patients were brought in. It was not right that a nurse should treat patients at a hospital unless by the direction of a qualified medical man. In fairness to the public it ought to be made known that it was not a general hospital in the ordinary sense of the term.

The jury returned a verdict of "Accidental death," and added that in their opinion there had been neglect on the part of the hospital.

*Second Case.*

A second case in which the Anti-Vivisection Hospital was concerned was with regard to the death of a child aged thirteen months, who received fatal burns on December 13. The parents—named Good—live at Knowsley Road, Battersea.

The father of the child said that after his wife had taken her to the hospital on two or three mornings she was told to come twice a day—at 9 A.M. and 8 P.M. After nine days she was told she would have to get a doctor and a nurse. The parish doctor gave an order for the child's admission to the infirmary, where she was taken on December 21. She died on Christmas morning.

The mother said she took the child to the hospital, where the matron said she ought to be admitted, but there was no bed. The witness took her daily, but did not see a doctor till the third day. She waited three hours on one occasion. A nurse dressed the injuries.

A card which had been sent from the hospital was referred to by the Coroner. It was filled in with particulars of the case. The witness said those particulars were all written by the nurse on the fourth day of attendance. She was told to put her initials at the bottom, which she did, though the card was not read to her.

The Coroner pointed out that according to the card itself the initialling was a sign that the person who brought the patient understood the directions given at the hospital.

Dr. J. B. Neal, Medical Superintendent of Wandsworth Infirmary, said the child should have been taken into an institution earlier, in which case she would have had a much better chance of recovery.

Dr. Munro, Acting Medical Officer at the Anti-Vivisection Hospital, said he saw the child when she was brought in on December 13. The nurse drew his attention to anything particularly serious. He was at the hospital from 9 till 11.

The Coroner : It is not fair that a diagnosis should be made by a nurse.

Dr. Munro : It is made by me.

I understood you to say that the nurse drew your attention to serious cases.—I withdraw that. The fact is, I see the cases. I am supposed to see every case that comes into the hospital.

The witness further stated that the mother was recommended to take the child to the Children's Hospital, as there was no accommodation for very young children at his hospital.

When informed that the mother had stated there was no doctor there on several occasions the witness said, "I am always there in the morning."

The Coroner reminded him that on other occasions there had been no doctor in attendance, and mentioned the case of Styan, which had been dealt with that afternoon. On that the witness said there were certain hours during which he was not in. As far as he was aware he saw the child daily.

A woman who had accompanied the mother to the hospital generally corroborated Mrs. Good's evidence.

The Coroner, summing up, said that the hospital practice everywhere was that when a nurse saw anything was happening she called the doctor at once, but, apart from that, in any properly equipped institution doctors attended and nurses acted under their directions. According to the evidence, on only one out of the ten occasions that the child was taken to the hospital did the doctor see it. It should be widely known what the treatment might be if people went to the Anti-Vivisection Hospital. It was for the governors of this hospital to consider these facts and their responsibility. If they thought to run an institution which on inquiry presented these results as to the treatment which patients receive, it would end not in the kind of case they were hearing, but in something much more grave. It was the duty of the governors to take very strong action to put their house in order.

The jury returned a verdict of "Accidental death," and expressed the opinion that there had been great neglect on the part of the authorities of the Anti-Vivisection Hospital.

The case was fully reported in the *Times* and *Telegraph* of the 29th ultimo.

